# Variable Weighted Ordered Subset Image Reconstruction Algorithm

**DOI:** 10.1155/IJBI/2006/10398

**Published:** 2006-10-12

**Authors:** Jinxiao Pan, Tie Zhou, Yan Han, Ming Jiang

**Affiliations:** ^1^ LMAM, School of Mathematical Sciences, Peking University, Beijing 100871, China; ^2^ Department of Mathematics, North University of China, Taiyuan, Shanxi 030051, China

## Abstract

We propose two variable weighted iterative reconstruction algorithms (VW-ART and VW-OS-SART) to improve the algebraic reconstruction technique (ART) and simultaneous algebraic reconstruction technique (SART) and establish their convergence. In the two algorithms, the weighting varies with the geometrical direction of the ray. Experimental results with both numerical simulation and real CT data demonstrate that the VW-ART has a significant improvement in the quality of reconstructed images over ART and OS-SART. Moreover, both VW-ART and VW-OS-SART are more promising in convergence speed than the ART and SART, respectively.


Hindawi Publishing Corporation
International Journal of Biomedical Imaging
Volume 2006, Article ID 10398, Pages 1–7
DOI 10.1155/IJBI/2006/10398

### Variable Weighted Ordered Subset Image Reconstruction Algorithm

**Jinxiao Pan,[1,2] Tie Zhou,[1] Yan Han,[2] and Ming Jiang[1]**

[1] *LMAM, School of Mathematical Sciences, Peking University, Beijing 100871, China*
[2] *Department of Mathematics, North University of China, Taiyuan, Shanxi 030051, China*

Received 17 February 2006; Revised 25 May 2006; Accepted 18 July 2006

Recommended for Publication by Seung Wook Lee

We propose two variable weighted iterative reconstruction algorithms (VW-ART and VW-OS-SART) to improve the algebraic reconstruction technique (ART) and simultaneous algebraic reconstruction technique (SART) and establish their convergence. In the two algorithms, the weighting varies with the geometrical direction of the ray. Experimental results with both numerical simulation and real CT data demonstrate that the VW-ART has a significant improvement in the quality of reconstructed images over ART and OS-SART. Moreover, both VW-ART and VW-OS-SART are more promising in convergence speed than the ART and SART, respectively.

Copyright © 2006 Jinxiao Pan et al. This is an open access article distributed under the Creative Commons Attribution License, which permits unrestricted use, distribution, and reproduction in any medium, provided the original work is properly cited.

### 1. INTRODUCTION

Many image reconstruction problems can be modeled by the following linear system:

$$y = Rx, \tag{1}$$

where $y = (y_1, y_2, \dots, y_n)^T \in \mathbb{R}^n$ is the observed data, $n$ is the number of the projection datum, $x = (x_1, x_2, \dots, x_m)^T \in \mathbb{R}^m$ is the original image ($T$ denotes the transpose of a matrix, $\mathbb{R}$ is the real number field, and $m$ is the number of pixel, namely, image array is $\sqrt{m} \times \sqrt{m}$) and $R = (r_{ij})$ is an $n \times m$ matrix. Image reconstruction is to estimate the original image $x$ from the observed data $y$. Since (1) is usually ill-conditioned and the data in practice are noisy and very large, a solution by direct method involving the matrix $R$ is infeasible [1]. Instead, some iterative methods, such as the algebraic reconstruction technique (ART) [2], simultaneous algebraic reconstruction technique (SART) [3], and expectation maximization (EM) algorithms [4], have been developed for image reconstruction. The iterative algorithm is a promising approach to achieve a better image quality in CT. However, limitations exist with respect to the required computation time.

In order to accelerate the convergence of iterative algorithms in image reconstruction, several improvements were designed based on the SART and EM algorithms. One remarkable technique is the ordered subset (OS) (also called block iterative (BI)) versions of the simultaneous schemes [5]. There are some guidelines to be considered [6] in the selection of subsets and blocks, but the relaxation parameters are fixed in numerical implementations [7, 8]. In this paper, we propose variable weighted OS-SART (VW-OS-SART) and variable weighted ART (VW-ART) algorithms. Since the weights are considered in these subsets, the reconstructed images by VW-ART are better than those by OS-SART, VW-OS-SART, and ART. The reconstruction speeds of these algorithms are faster than those by OS-SART and those of ART.

The structure of the manuscript is as follows. In Section 2, we introduce a method for subset partition and corresponding weights selection. In Section 3, we derive the VW-OS-SART and VW-ART algorithms. In Section 4, we discuss the convergence property of the proposed algorithms. In Section 5, we apply our algorithms to simulated and practical data. In Section 6, we discuss some relevant issues and conclude the paper.

### 2. SUBSETS PARTITION AND WEIGHTS SELECTION

The main idea of ordered subset method is to partition the dataset into several subsets. It is crucial to find an effective way for the partitioning. In each iteration, we hope that the image is improved as much as possible with a lower computational cost. Hence, each subset should contain as much complementary tomographic information as possible and have a relatively small size for computational balance. One of the natural subset partitions is formulated by dividing the projection data according to the projection angles. For example, for system (1), the 2D image array is $\sqrt{m}\times\sqrt{m}$, then there are $n/\sqrt{m}$ projections and $\sqrt{m}$ identical detector bins per projection. The projection angle interval $[0,\ 2\pi]$ can be divided into $T$ subintervals of the same length ($T$ is the number of the subintervals):

$$\left[0,\frac{4\pi}{T}\right),\left[\frac{4\pi}{T},\frac{8\pi}{T}\right),\dots,\left[\frac{2(T-1)\pi}{T},2\pi\right),\left[\frac{2\pi}{T},\frac{6\pi}{T}\right),$$
$$\left[\frac{6\pi}{T},\frac{10\pi}{T}\right),\dots,\left[\frac{(2T-1)\pi}{T},2\pi\right)\cup\left[0,\frac{2\pi}{T}\right). \tag{2}$$

There are $2n/T\sqrt{m}$ the observed projection angles in each subinterval.

The projections are sequenced according to geometrical direction of the ray. The projection data of rays are sequenced according to geometrical position. Denote the index of projection datum by $B = \{1, 2, \dots, n\}$. Then we partition the index set $B$ into $T$ nonempty subsets

$$B_t = \{i_1^t, i_2^t, \dots, i_{n(t)}^t\},$$

$$B = \{1, 2, \dots, n\} = \bigcup_{1\le t\le T} B_t. \tag{3}$$

Each $B_t$ contains all the indices of projection data in the $t$th angle subinterval in (2). The subsets $B_t$ are not necessarily disjoint.

The weights of the all projection data in one projection angle are equal. We define

$$\mu_1(\alpha) = \frac{T}{2\pi}r,\quad \mu_T(\alpha) = 1-\mu_1(\alpha),\quad t = 1, 0\le r\le\frac{2\pi}{T},$$
$$\mu_t(\alpha)=\frac{T}{2\pi}r,\quad \mu_{[T/2+t-1]}(\alpha)=1-\mu_t(\alpha),\quad t\ne 1, 0\le r\le\frac{2\pi}{T},$$
$$\mu_t(\alpha) = 2-\frac{T}{2\pi}r,\quad \mu_{[T/2+t]}(\alpha) = 1-\mu_t(\alpha),\quad r>\frac{2\pi}{T}, \tag{4}$$

where $\alpha = (4\pi/T)(t-1)+r$, ($t = 1, 2, \dots, T/2$, $0\le r < 4\pi/T$). $\mu_{B_t}(y_i)$ is the weighted of the $i$th projection datum $y_i$ in $B_t$, then $\mu_{B_t}(y_i) = \mu_t(\alpha)$, where $\alpha$ is the projection angle of $y_i$.

See Figure 1 for the geometrical interpretation of the weights selection. In each projecting angle subinterval, the weights locate on the two-segment line. Therefore the projection data index set $B$ is divided into $T$ subsets $\{B_1, B_2, \dots, B_T\}$, and the weight corresponding to each subset is given by $\mu_{B_t}(y_i)$ which satisfies

$$\sum_{t=1}^{T}\mu_{B_t}(y_i) = 1,\quad i = 1, 2, \dots, m. \tag{5}$$

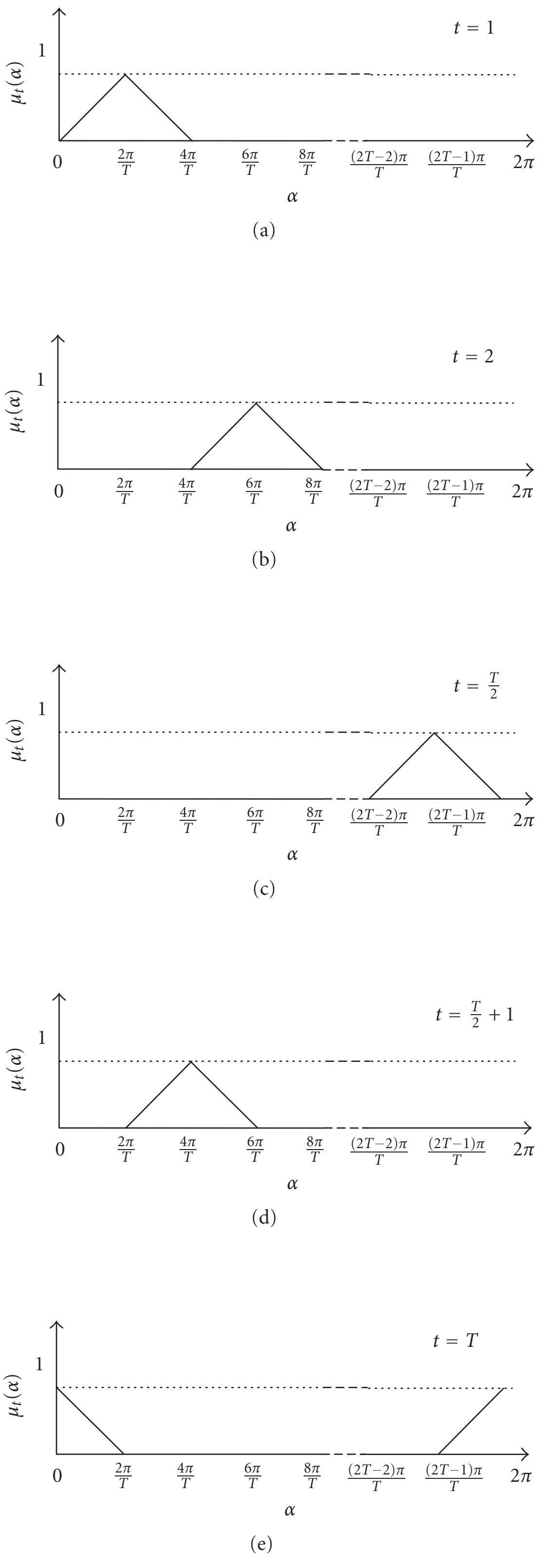

Figure 1: Geometrical interpretation of the weights selection in formula (4), $\alpha$ denote projection angle, $\mu_t(\alpha)$ denote the weighted of the projection data at $\alpha$ projection angle in $t$th subinterval, $\mu_t(\alpha) = 0$, when $t\ne[\alpha/4\pi/T]+1$, ($[x]$ denote the integral part of $x$).

### 3. VARIABLE WEIGHTED VERSION OF OS-SART AND VARIABLE WEIGHTED ART ALGORITHMS

Let $V$ and $W$ be two positive definite diagonal matrices of order $m$ and $n$, respectively. The following general Landweber scheme was studied in [1]:

$$x^{(l+1)} = x^{(l)} + \lambda_l V^{-1} R^T W\left(y - Rx^{(l)}\right), \tag{6}$$

where $\lambda_l > 0$ is the relaxation coefficient in the $l$th iteration. When

$$\begin{aligned} V &= \operatorname{diag}\left\{\sum_{i=1}^{n} r_{i1}, \sum_{i=1}^{n} r_{i2}, \ldots, \sum_{i=1}^{n} r_{im}\right\}, \\ W &= \operatorname{diag}\left\{\frac{1}{\sum_{j=1}^{m} r_{1j}}, \frac{1}{\sum_{j=1}^{m} r_{2j}}, \ldots, \frac{1}{\sum_{j=1}^{m} r_{nj}}\right\}, \end{aligned} \tag{7}$$

then (6) is the SART [5]

$$x_j^{(l+1)} = x_j^{(l)} + \lambda_l \frac{1}{\sum_{i=1}^{n} r_{ij}} \sum_{i=1}^{n} \frac{r_{ij}}{\sum_{j=1}^{m} r_{ij}}\left(y_i - R^i x^{(l)}\right), \tag{8}$$

where $R^i$ is the $i$th row of $R$.

According to the partitioning and weighting strategies proposed in Section 2, the variable weighted version of the OS-SART algorithm (VW-OS-SART) can be written as

$$\begin{aligned} x_j^{(l+1)} &= x_j^{(l)} + \lambda_l \frac{1}{\sum_{i\in B_{[l]}} r_{ij}\mu_{B_{[l]}}(y_i)} \\ &\quad \bullet \sum_{i\in B_{[l]}} \mu_{B_{[l]}}(y_i) \frac{r_{ij}}{\sum_{j=1}^{m} r_{ij}}\left(y_i - R^i x^{(l)}\right), \end{aligned} \tag{9}$$

where $[l] = l \bmod (T) + 1$ for $l \geq 0$. The iteration process from $l = \nu T$ to $l = (\nu + 1)T$ is called one cycle, in which each subset $B_t$ is visited exactly once. When

$$\begin{aligned} V &= \operatorname{diag}\left\{\sum_{i\in B_{[l]}} r_{i1}\mu_{B_{[l]}}(y_i),\right. \\ &\qquad \left.\sum_{i\in B_{[l]}} r_{i2}\mu_{B_{[l]}}(y_i), \ldots, \sum_{i\in B_{[l]}} r_{im}\mu_{B_{[l]}}(y_i)\right\}, \\ W &= \operatorname{diag}\left\{\frac{\mu_{B_{[l]}}(y_1)}{\sum_{j=1}^{m} r_{1j}}, \frac{\mu_{B_{[l]}}(y_2)}{\sum_{j=1}^{m} r_{2j}}, \ldots, \frac{\mu_{B_{[l]}}(y_n)}{\sum_{j=1}^{m} r_{nj}}\right\} \end{aligned} \tag{10}$$

then (6) is the same as (9).

When $V = I$, $W = \operatorname{diag}\{\mu_1/\sum_{j=1}^{m} r_{1j}^2, \mu_2/\sum_{j=1}^{m} r_{2j}^2, \ldots, \mu_n/\sum_{j=1}^{m} r_{nj}^2\}$, (6) becomes the ART [1]. The variable weighted version of this algorithm, called VW-ART, can be written as

$$x_j^{(l+1)} = x_j^{(l)} + \lambda_l \frac{r_{ij}\mu_i}{\sum_j r_{ij}^2}\left(y_i - R^i x^{(l)}\right), \tag{11}$$

where $i = l \bmod (n) + 1$, and

$$\mu_i = \begin{cases} \mu_i^{(1)} = \max\limits_{1\leq t\leq T/2} \{\mu_{B_t}(y_i)\} \\ \qquad 2\nu n \leq l < (2\nu + 1)n, \nu = 0, 1, 2, \ldots, \\ \mu_i^{(2)} = \max\limits_{T/2+1\leq t\leq T} \{\mu_{B_t}(y_i)\} \\ \qquad (2\nu + 1)n \leq l < 2(\nu + 1)n, \nu = 0, 1, 2, \ldots. \end{cases} \tag{12}$$

### 4. CONVERGENCE ANALYSIS

Let

$$\begin{aligned} R_t &= \begin{pmatrix} \mu_{B_t}\left(y_{i_1^t}\right) R^{i_1^t} \\ \vdots \\ \mu_{B_t}\left(y_{i_{n(t)}^t}\right) R^{i_{n(t)}^t} \end{pmatrix}_{n(t)\times m}, \\ W_t &= \begin{pmatrix} W^{i_1^t} & & \\ & \ddots & \\ & & W^{i_{n(t)}^t} \end{pmatrix}_{n(t)\times n(t)}, \\ y^{(t)} &= \begin{pmatrix} y_{i_1^t}\mu_{B_t}\left(y_{i_1^t}\right) \\ \vdots \\ y_{i_{n(t)}^t}\mu_{B_t}\left(y_{i_{n(t)}^t}\right) \end{pmatrix} \in \mathbb{R}^{n(t)}, \end{aligned} \tag{13}$$

where $R^i$ and $W^i$ are the $i$th row of $R$ and the $i$th diagonal element of $W$, respectively. Denote

$$C = \begin{pmatrix} R_1 \\ R_2 \\ \vdots \\ R_T \end{pmatrix}, \qquad b = \begin{pmatrix} y^{(1)} \\ y^{(2)} \\ \vdots \\ y^{(T)} \end{pmatrix}, \tag{14}$$

where $C = (c_{ij})_{(2n-T\sqrt{m})\times m}$, $b$ is a $2n - T\sqrt{m}$ dimension vector, then we have a linear algebraic system

$$Cx = b. \tag{15}$$

Equation (1) is consistent if and only if (15) is consistent, and they have exactly the same solution set. The OS version of the Landweber scheme of (15) can be written as

$$\begin{aligned} x^{(l+1)} &= x^{(l)} + \lambda_l V^{-1} C_{[l]}^T W_{[l]}\left(b_{[l]} - C_{[l]} x^{(l)}\right) \\ &= x_j^{(l)} + \lambda_l \frac{1}{\sum_{i\in B_{[l]}} r_{ij}\mu_{B_{[l]}}(y_i)} \\ &\quad \bullet \sum_{i\in B_{[l]}} \mu_{B_{[l]}}(y_i) \frac{r_{ij}}{\sum_{j=1}^{m} r_{ij}}\left(y_i - R^i x^{(l)}\right), \end{aligned} \tag{16}$$

where $W_{[l]} = \operatorname{diag}\{\sum_{j=1}^{m} c_{1j}, \sum_{j=1}^{m} c_{2j}, \ldots, \sum_{j=1}^{m} c_{(2n-T\sqrt{m})j}\}$. Formula (16) is the same as the VW-OS-SART formula (9).

Similar to that in [5], we get the following convergence results.

**Theorem 1.** *If $0 < \lambda_l < 2$, for all $l \geq 0$, $\sum_l \lambda_l = \infty$, and $\lim_{l\to\infty}\lambda_l = 0$, then the sequence $\{x^{(l)}\}$ generated by (9) converges to $x^{(*)} + P_V x^{(0)}$ even with inconsistent data. Here, $P_V$ is the orthogonal projection from $\mathbb{R}^m$ to the kernel of $R$ with respect to the inner product $\langle\cdot,\cdot\rangle_V$.*

VW-ART algorithm is a special ART algorithm, where the relaxation parameters $\{\lambda_l\}$ are special sequence, namely, the relaxation parameters are $\lambda_l\mu_i$ (where $\mu_i \leq 1$). If the sequence $\{x^{(l)}\}$ generated by the ART algorithm converges, the sequence$\{x^{(l)}\}$ generated by the VW-ART algorithm (11) converges, too. That is the following theorem.

**Theorem 2.** *If* $0 < \lambda_l \mu_l < 2$, *for all* $l \geq 0$, $\sum_l \lambda_l = \infty$, *and* $\lim_{l \to \infty} \lambda_l = 0$, *then the sequence* $\{x^{(l)}\}$ *generated by* (11) *converges to* $x^{(*)} + P_V x^{(0)}$.

In order to study the speed of convergence, we call finishing one VW cycle 2 iteration numbers, because the consuming time of one VW-OS-SART cycle is almost twice as that of one OS-SART cycle ($T/2$ ordered subsets), and the time of one VW-ART cycle is twice as that of one ART cycle [9]. Namely, the iteration numbers in variable weighted algorithm are always even.

### 5. EXPERIMENT RESULTS

To test the performance of the VW-OS-SART, and compare it with OS-SART and VW-ART, we code all of them in the programming language C on a PC (RAM: 256 MB, CPU: Intel P4 2.8 GHz) under Windows XP operating system. The image array is 256 × 256, with 256 gray levels. Projections are simulated (or obtained in experiment) over 360° with the uniform sampling scheme. There are totally 360 projections, 256 identical detector bins per projection. Pseudo random noises satisfying Gauss distribution are added to the Shepp-Logan phantom data, with variance being 2.5% of image mean, and the relaxation sequence $\lambda_l = 2$. The numbers of subsets $T = 72$, each $B_t$ containing nine projections, that is, $m = 256^2$, $n = 256 \times 360$. The weights are computed as in Section 1. For example, if $y$ is one of the 256 projections with the projection degree 1°, then $\mu_{A_1}(y) = 0.2, \mu_{A_{72}}(y) = 0.8$; if $y$ is one of the 256 projections with the projection degree 5°, then $\mu_{B_1}(y) = 1$; if $y$ is a projection with the projection degree 8°, then $\mu_{B_1}(y) = 0.4, \mu_{B_{37}}(y) = 0.6$; if $y$ is one of the 256 projections with the projection degree 10°, then $\mu_{B_{37}}(y) = 1$.

In Figure 2, we present a Shepp-Logan phantom and the images reconstructed by the above algorithms (the initial value of iterative reconstruction is $x^{(0)} = (0, 0, \ldots, 0)$). It can be seen from Figure 2 that all the reconstructed images by VW-ART are better than those by OS-SART, VW-OS-SART (relaxation parameter is 2) and ART. The reconstructed speeds of VW-OS-SART are faster than those by OS-SART; the reconstruction speeds of VW-ART are faster than those of ART (see Figure 3).

Since the true image is known in the Shepp-Logan phantom experiment, to quantify the performance of the VW-OS-SART and VW-ART formulas, the mean square error is computed from the images in the CT phantom experiment. In Figure 3, we present the difference of the mean square error of the reconstructed images by ART, VW-ART, OS-SART, and VW-OS-SART.

In the practical data experiments we use a cylindrical object of 80 mm in diameter, with 14 apertures of different diameters inside, and scan with X-ray generator with 220 KVp and 10 mA. The whole scan time is 1.5 second. The distance between the focus and the detector is 1.000 mm; the distance between the center of rotation and the detector is 50 mm. Figure 4(a) shows the image profile of the object, Figures 4(b)–4(e) show the images reconstructed via different algorithms ($\lambda_l = 0.15$, computation time 600 seconds).

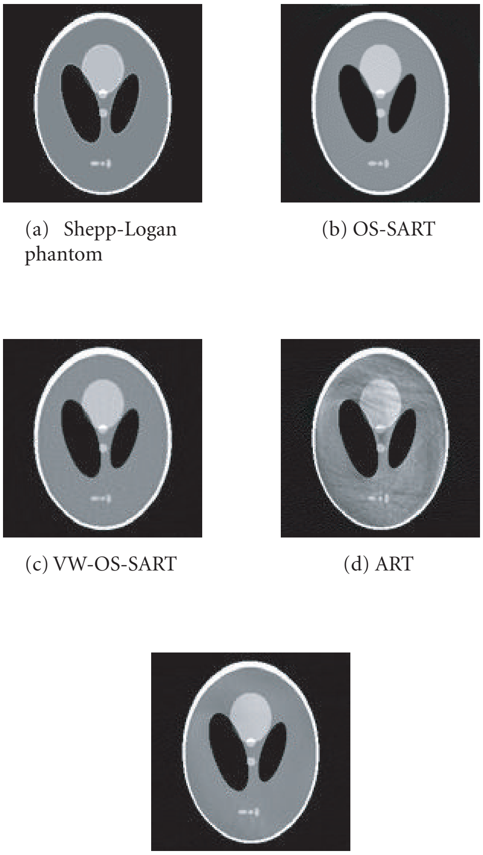

(a) Shepp-Logan phantom

(b) OS-SART

(c) VW-OS-SART

(d) ART

(e) VW-ART

Figure 2: Phantom experiment. The gray range is from 0 to 255. (a) is the Shepp-Logan phantom. (b)–(e) are reconstructed images with the above algorithms.

The images reconstructed by the VW-ART (Figure 4(e)) are better than those reconstructed by the OS-SART, the VW-OS-SART, and the ART (Figures 4(b)–4(d)) in quality. The criterion of comparison is the resolution of images, namely, whether the apertures can be detected.

### 6. DISCUSSIONS AND CONCLUSION

In our experiments, we have found that for both VW-OS-SART and VW-ART, the partition of ordered subset affects the quality of reconstruction image. And the selection of weights for each projection is crucial to variable weighted algorithms, and it can be a piece-wise linear function, Gaussian function and so on. The convergence conditions for VW-OS-SART and VW-ART are guidelines for the selection of the relaxation parameters. The relaxation parameters in ART algorithm and VW-ART algorithm are generally smaller than those in SART algorithm and VW-OS-SART algorithm.

The partition of the subsets is generally based on the geometrical relationship of projections. The weights selection is given by linearity in this paper, and it can be given by an exponential as well, which is similar to ordered subsets method,

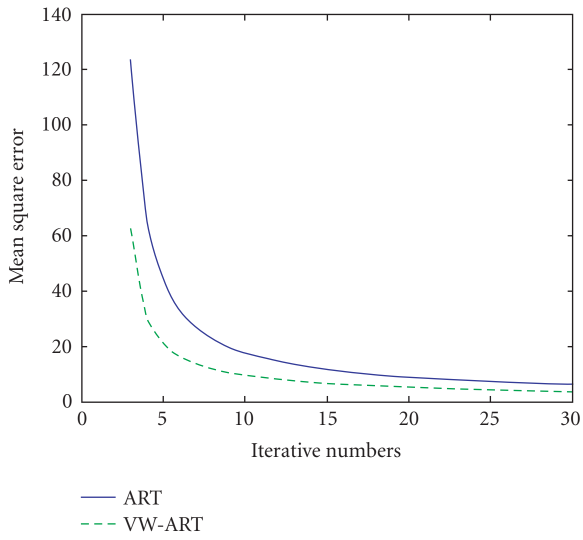

(a) ART & VW-ART

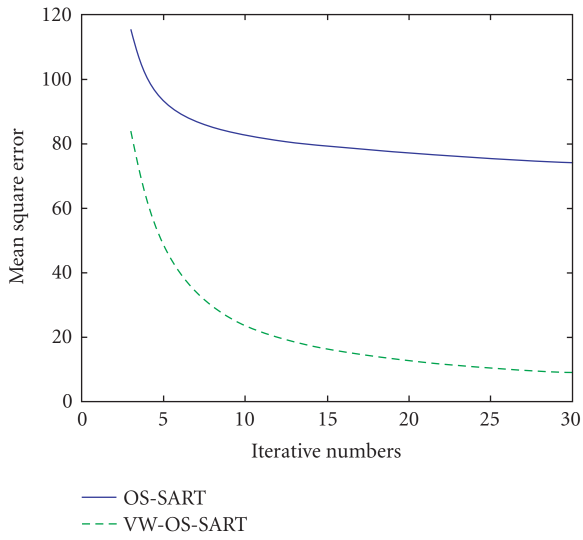

(b) OS-SART & VW-OS-SART

FIGURE 3: Plot of mean square error as a function of iterative number in the Shepp-Logan phantom experiments.

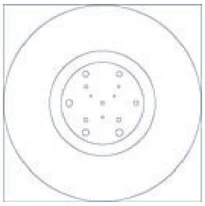

(a) Image profile

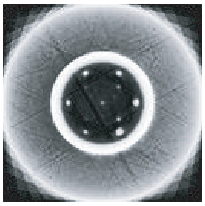

(b) OS-SART

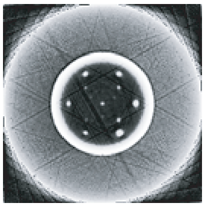

(c) VW-OS-SART

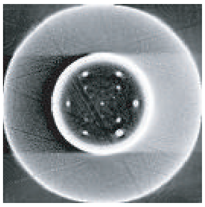

(d) ART

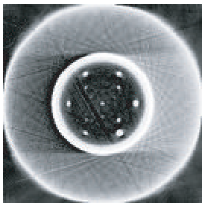

(e) VW-ART

FIGURE 4: Reconstructed images by different algorithms using the practical data. The gray range is from 0 to 255.

generally, "*increasing the number of the subsets accelerates iterative convergence, but there is a point beyond which image quality degrades due to lack of either tomographic or statistical information within subsets*" [6]. The number of subsets may affect the convergence of the algorithm. Instead of partitioning the data in the projection domain, another way is to partition the pixel set in the image domain, which is under investigation.

In conclusion, we have proposed the VW-OS-SART and VW-ART algorithms and established their convergence. The proposed algorithms have been evaluated with Shepp-Logan phantom and practical data. In VW-OS-SART and VW-ART algorithms, the sum of the weighted of each projection data is 1, the weighting varies with the geometrical direction of the ray, the weight and partitioning procedures are intuitively reasonable and easy to be implemented. We use a weighted partitioning scheme such that each subset contains equal-spaced angle X-rays only, then these algorithms can have a significant improvement in the quality of reconstructed images over ART and SART, the experimental results on both digital phantom and real CT data have demonstrated that the VW-ART has a significant improvement in the quality of reconstructed images over ART and SART; both VW-ART and VW-OS-SART are more promising in convergence speed than ART and SART, respectively.

### APPENDIX

### PROOF OF THEOREM 1

Let $x^{(*)}$ be the minimal $V$ norm solution of the problem, where the $V$ norm is defined as $\|x\|_V^2 = \langle x, x\rangle_V = \langle Vx, x\rangle$, and $P_V$ is the orthogonal projection from $\mathbb{R}^m$ to the kernel of $R$ (the kernel of $C$ is identical with that of $R$) with respect to the inner product $\langle \cdot, \cdot\rangle_V$, and $\|R\|_{V,W}$ is the operator norm from $\mathbb{R}^m$ (with $\langle \cdot, \cdot\rangle_V$) to $\mathbb{R}^n$(with $\langle \cdot, \cdot\rangle_W$). The following lemma is proved in [1].

**Lemma A.1.** *Assume that there exists* $\rho > 0$ *such that* $\|R_t\|_{V,W_t} \le \rho$ *for* $t = 1, 2, \ldots, T$ *and* $0 \le \rho^2 \lambda_l \le 2$, *if* (15) *is consistent and*

$$\sum_l \min\left(\rho^2 \lambda_l, 2 - \rho^2 \lambda_l\right) = \infty, \tag{A.1}$$

*then the sequence* $\{x^{(l)}\}$ *generated by* (16) *converges to* $x^{(*)} + P_V x^{(0)}$. *If*

$$\sum_l \lambda_l = \infty, \qquad \lim_{l \to \infty} \lambda_l = 0, \tag{A.2}$$

*then the sequence* $\{x^{(l)}\}$ *generated by* (16) *converges to* $x^{(*)} + P_V x^{(0)}$, *even if* (15) *is inconsistent.*

Because (1) is consistent if and only if (15) is consistent, the formula (16) is the same as formula (9). The following lemma of Theorem 1 is proved in [1].

**Lemma A.2.** *Assume that there exists* $\rho > 0$ *such that* $\|R_t\|_{V,W_t} \le \rho$ *for* $t = 1, 2, \ldots, T$ *and* $0 \le \rho^2 \lambda_l \le 2$, *if* (1) *is consistent and*

$$\sum_l \min\left(\rho^2 \lambda_l, 2 - \rho^2 \lambda_l\right) = \infty, \tag{A.3}$$

*then the sequence* $\{x^{(l)}\}$ *generated by* (9) *converges to* $x^{(*)} + P_V x^{(0)}$. *If*

$$\sum_l \lambda_l = \infty, \qquad \lim_{l \to \infty} \lambda_l = 0, \tag{A.4}$$

*then the sequence* $\{x^{(l)}\}$ *generated by* (9) *converges to* $x^{(*)} + P_V x^{(0)}$, *even if* (1) *is inconsistent.*

To establish the convergence of the proposed algorithms, we estimate $\|C\|_{V,W}$ with the corresponding $V$ and $W$ as follows:

$$\begin{aligned}
\|Cx\|_W^2 &= \sum_{i=1}^{n} \frac{1}{\sum_j c_{ij}} \left| \sum_j c_{ij} x_j \right|^2 \\
&= \sum_{i=1}^{n} \frac{1}{\sum_{j=1}^{m} \mu_i^{(1)} r_{ij} + \sum_{j=1}^{m} \mu_i^{(2)} r_{ij}} \\
&\quad \times \left| \sum_{j=1}^{m} \mu_i^{(1)} r_{ij} x_j + \sum_{j=1}^{m} \mu_i^{(2)} r_{ij} x_j \right|^2 \\
&= \sum_{i=1}^{n} \frac{1}{\sum_{j=1}^{m} r_{ij}} \left| \sum_j r_{ij} x_j \right|^2 \\
&= \sum_{i=1}^{n} \left( \sum_{j=1}^{m} r_{ij} \left| \sum_{j=1}^{m} \frac{r_{ij}}{\sum_{j=1}^{m} r_{ij}} x_j \right|^2 \right) \\
&\le \sum_{i=1}^{n} \left( \sum_{j=1}^{m} r_{ij} \left( \sum_{j=1}^{m} \frac{r_{ij}}{\sum_{j=1}^{m} r_{ij}} \left| x_j \right|^2 \right) \right) \\
&= \sum_{j=1}^{m} \left( \sum_{i=1}^{n} r_{ij} \left| x_j \right|^2 \right) = \|x\|_V,
\end{aligned} \tag{A.5}$$

where the inequality is according to the convexity of the function $t \mapsto t^2$. Therefore, $\|C\|_{V,W} \le 1$, we can choose $\rho$ to be 1, and get the convergence result.

### ACKNOWLEDGMENTS

This work is partially supported by National Science Foundation of China (60372024, 60372073, 60325101, 60532080), the key Project of Chinese Ministry of Education (No. 306017). Jinxiao Pan is partially supported by Shanxi Province Science Foundation of China (20051042) and Beijing Academy of Science and Technology. Tie Zhou and Ming Jiang are partially supported by Engineering Research Institute, Peking University. Tie Zhou is partially supported by Center for Computational Science & Engineering, Peking University.

### REFERENCES

[1] M. Jiang and G. Wang, "Convergence studies on iterative algorithms for image reconstruction," *IEEE Transactions on Medical Imaging*, vol. 22, no. 5, pp. 569–579, 2003.

[2] R. Gordon, R. Bender, and G. T. Herman, "Algebraic reconstruction techniques (ART) for three-dimensional electron microscopy and X-ray photography," *Journal of Theoretical Biology*, vol. 29, no. 3, pp. 471–481, 1970.

[3] A. H. Andersen and A. C. Kak, "Simultaneous algebraic reconstruction technique (SART): a superior implementation of the ART algorithm," *Ultrasonic Imaging*, vol. 6, no. 1, pp. 81–94, 1984.

[4] L. A. Shepp and Y. Vardi, "Maximum likelihood reconstruction for emission tomography," *IEEE Transactions on Medical Imaging*, vol. 1, no. 2, pp. 113–122, 1982.

[5] M. Jiang and G. Wang, "Development of iterative algorithms for image reconstruction," *Journal of X-Ray Science and Technology*, vol. 10, no. 1-2, pp. 77–86, 2002.

[6] D. J. Kadrmas, "Statistically regulated and adaptive EM reconstruction for emission computed tomography," *IEEE Transactions on Nuclear Science*, vol. 48, no. 3, part 2, pp. 790–798, 2001.

[7] H. H. Bauschke and J. M. Borwein, "On projection algorithms for solving convex feasibility problems," *SIAM Review*, vol. 38, no. 3, pp. 367–426, 1996.

[8] Y. Censor and S. A. Zenios, *Parallel Optimization Theory, Algorithm, and Applications*, Oxford University Press, New York, NY, USA, 1997.

[9] G. Wang and M. Jiang, "Ordered-subset simultaneous algebraic reconstruction techniques (OS-SART)," *Journal of X-Ray Science and Technology*, vol. 12, no. 3, pp. 169–177, 2004.

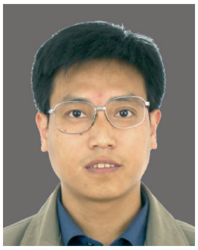

**Jinxiao Pan** is a Professor with College of Science, Department of Mathematics, North University of China. He obtained his B.S. degree in probability and statistics from Xi'an Jiaotong University, Xi'an, Shaanxi, China in 1994, and Ph.D. degree in information engineering from North University of China, Taiyuan, Shanxi, China in 2003. He is a Postdoctoral Fellow with School of Mathematical Science, Peking University from 2004 to 2006. His interests are image reconstruction algorithms and image restoration.

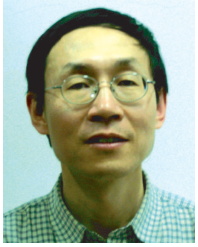

**Tie Zhou** is an Associate Professor of computational mathematics at Peking University. He received his Ph.D. degree in mathematics from Peking University (1994). He received his M.S. degree in mathematics from Peking University in 1990. He received his B.A. degree in mathematics from Peking University in 1985. His research interests include biomedical imaging, numerical partial differential equations, and computational fluid dynamics.

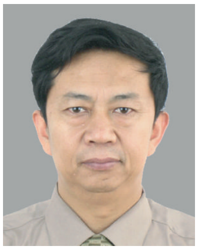

**Yan Han** is a Professor with School of Information and Communication Engineering, Department of Information Engineering, North University of China. He received his B.S. degree in testing technology from North University of China, Taiyuan Shanxi, China in 1987, and Ph.D. degree in signal and information processing from Beijing University of Science and Technology, Beijing, China in 1998. His interests are nondestructive test, computed tomography, and image processing.

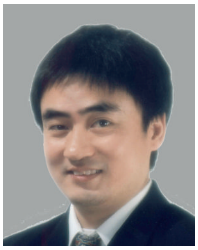

**Ming Jiang** is a Professor with School of Mathematical Science, Department of Information Science, Peking University. He obtained his B.S. and Ph.D. degrees in nonlinear analysis from Peking University in 1984 and 1989, respectively. He was an Associate Professor with Department of Applied Mathematics, Beijing Institute of Technology from 1989 to 1995; Postdoctoral Fellow with Microprocessor Laboratory, International Centre for Theoretical Physics, Italy, from 1996 to 1997; Visiting Professor and Associate Director of CT/Micro-CT Laboratory, Department of Radiology, University of Iowa, USA, from 2000 to 2002. His interests are image reconstruction, restoration, and analysis.

